# Recurrent cardiac events in patients with idiopathic ventricular fibrillation, excluding patients with the Brugada syndrome

**DOI:** 10.1186/1741-7015-3-1

**Published:** 2005-01-01

**Authors:** Jean Champagne, Peter Geelen, François Philippon, Pedro Brugada

**Affiliations:** 1 Cardiovascular Center, OLV Ziekenhuis, Moorselbaan 164, 9300 Aalst, Belgium; 2 Quebec Heart Institute, Laval Hospital, 2725, Chemin Ste-Foy, Quebec City, Quebec, Canada

## Abstract

**Background:**

The recurrence of cardiac events in patients with idiopathic ventricular fibrillation (VF) *excluding *patients with the Brugada syndrome is unclear since this entity remains present in previous studies.

**Methods:**

Since 1992, 18 patients (72% male) with idiopathic VF out of 455 ICD implants were treated with an implantable cardioverter defibrillator (ICD). The mean age at first ICD implantation was 42 ± 14 years. Brugada syndrome, as well as other primary electrical diseases (e.g. long QT), were systematically excluded in all patients by the absence of the typical electrocardiogram (ST elevation in the right precordial leads) at rest and/or after pharmacological tests (ajmaline, flecainide, or procainamide). Recurrence of cardiac events was prospectively assessed.

**Results:**

During a mean follow-up period of 41 ± 27 months, VF recurrence with appropriate shock occurred in 7 patients (39%) covering a total of 27 shocks. The median time to first appropriate shock was 12 ± 9 months. There were no deaths. In the electrophysiological study, 39% of patients were inducible, but inducibility failed to predict subsequent arrhythmic events. Forty-four percent of patients suffered 21 inappropriate shocks, which were caused by sinus tachycardia, atrial arrhythmias or lead malfunction.

**Conclusion:**

Idiopathic ventricular fibrillation patients have a high recurrence rate of potentially fatal ventricular arrhythmias, *excluding *patients with the Brugada syndrome or other known causes. ICD prevents sudden cardiac death but inappropriate shocks remained a major issue in this young and active population.

## Background

Idiopathic ventricular fibrillation (VF) is defined as spontaneous ventricular fibrillation in the absence of any structural heart disease, including coronary artery disease, valvular heart disease, myocarditis, cardiomyopathy or electrophysiological diseases, with a well-defined cause, such as the long QT syndrome, the Brugada syndrome, ventricular pre-excitation (WPW), and drug intoxication [1]. The consensus statement of the joint steering committees of the UCARE registry of Europe and of the idiopathic VF registry of US reported in 1997 that patients with the Brugada syndrome should be considered a variant of idiopathic VF [1].

Brugada syndrome is characterized by a unique electrocardiographic (ECG) pattern of right bundle branch block with ST elevation in the right precordial leads. Transient forms of the disease in which the ECG normalizes for a period of time have been described. Administration of sodium channel blockers will unmask the abnormal ECG in patients with transient normalized ECGs [2,3]. The American Heart Association has recently proposed diagnostic criteria for the Brugada syndrome [4]. Moreover, the incidence of Brugada syndrome among patients with idiopathic VF remains unclear. The prevalence of a Brugada type ECG pattern was reported in 21% [5] and 24% [6] of idiopathic VF populations. However, on the basis of pharmacological tests, it has been speculated that up to 40% – 60% of patients diagnosed with idiopathic VF might actually suffer from the Brugada syndrome [7]. Since this is a new clinical entity, older publications probably classified some patients with Brugada syndrome as idiopathic VF, since intravenous administration of a sodium channel blocker to unmask a concealed form of the disease was not systematically performed. Our report describes the clinical and electrophysiological characteristics of consecutive sudden cardiac arrest survivors from idiopathic VF who received an ICD, and in whom electrical and structural heart diseases including Brugada syndrome, long QT, arrhythmogenic right ventricular dysplasia, and hypertrophic cardiomyopathy characterized by a high recurrence rate, were systematically excluded.

## Methods

### Patient population

The total population under investigation included 455 consecutive patients who received a third generation ICD, with stored electrograms capabilities for hemodynamically poorly tolerated ventricular tachyarrhythmias, from 1992 to 2000. Of these, 29 were initially diagnosed as idiopathic VF associated with a structurally normal heart, normal left and right ventricular ejection fraction and normal coronary arteries. During the follow-up, 11 patients developed a new diagnosis potentially explaining VF, and were excluded from the final analysis. Four had right ventricular dysplasia, 5 had Brugada syndrome with the typical ECG manifestations after a drug challenge, and 2 patients had long QT syndrome. A final diagnosis of idiopathic VF was made in the remaining 18 patients. All patients had survived either an episode of cardiac arrest due to VF (12 patients) or a syncopal episode associated with documented self-terminating polymorphic ventricular tachycardia or fibrillation (6 patients). In 15 patients, the documented VF was seen during daily activity (one episode during sustained effort) and in 3 patients, during night-time. Out of the 18 patients, 10 had a previous history of unexplained syncope.

### Definitions and investigation

Idiopathic VF was defined as VF in the absence of demonstrable cardiac abnormalities as previously reported [1]. Thus, we excluded other known causes of VT/VF such as WPW syndrome, congenital long QT syndrome, short-coupled *torsade de pointes*, catecholamine-induced polymorphic VT, and Brugada syndrome. No patient had a past history of ischemic heart disease, congestive heart failure or family history of unexpected sudden cardiac death. All patients had a normal physical examination, blood testing and exercise test. Structural heart disease was excluded in all by echocardiography, cardiac catheterization including right and left ventricular angiography and coronary angiography. Patients on anti-arhythmic therapy before their cardiac arrest were excluded from the study, as well as those with electrolyte disturbances, history of significant alcohol or drug abuse or prolonged QT_c_. Of the 18 patients, 10 patients had normal right ventricular endomyocardial biopsy and 8 patients had a normal cardiac magnetic resonance imaging. Moreover, in 5 patients, an ergonovine provocation test was performed to exclude coronary artery spasm. Brugada syndrome was systematically excluded in all patients by negative serial ECG recordings with the typical coved or saddle shaped-type ST-segment elevation in the right precordial leads. All patients received *iv *administration of a sodium channel blocker to unmask a concealed form of the disease and all tests were negative. Flecainide (2 mg/kg body weight) was used in 9 patients, procainamide (10 mg/kg body weight) in 4 patients, and ajmaline (0.7 mg/kg body weight) in 7 patients.

### Electrophysiological study

Programmed electrical stimulation was performed in all patients using up to 3 extra-stimuli at 3 different drive cycle lengths (600, 500, and 430 ms) delivered to the right ventricular apex. The coupling interval of the first 2 extra-stimuli was not shorter than 180 ms and not shorter than 200 ms for the third extra-stimuli. In case of non-inducibility, programmed stimulation was also performed from the right ventricular outflow tract. Sustained VF was defined as VF at a cycle length ≤ 200 ms and lasting ≥ 30 seconds or requiring immediate defibrillation. Sustained ventricular tachycardia (VT) was defined as VT lasting ≥ 30 seconds or requiring termination secondary to hemodynamic instability. Non-sustained VT was defined as >6 consecutive ventricular complexes or VT lasting <30 seconds.

### ICD implantation

All patients received a transvenous ICD with stored electrograms. Recorded episodes were reviewed and adjudicated as appropriate or inappropriate therapies. VF was defined at follow-up as consecutive ventricular beats recorded from the device at a cycle length of 240 msec or less. In all patients, the ICD was programmed with only 1 ventricular fibrillation detection zone at 180 bpm (333 msec). Only shock therapy was programmed. All patients were seen at follow-up in our centre.

### Statistical analysis

All data are expressed as mean ± standard deviation. Kaplan-Meier analysis was used to analyze time intervals until the first appropriate shock.

## Results

The study cohort consisted of 13 men and 5 women (Table 1), with a mean age at the first ICD implantation of 42 ± 14 years (range 20 to 70 years). Baseline electrocardiograms were normal in 16 patients. One patient had a left bundle branch block and 1 had a right bundle branch block. The mean QT_c _for the entire population was 410 ± 20 msec. All patients had a normal left ventricular ejection fraction (≥ 50%) without regional wall motion abnormalities. Mean left ventricular ejection fraction (EF) was 69 ± 8% (range 52 to 81%). No patient had significant coronary artery disease (defined as ≥ 50% stenosis). Microscopic examination of right ventricular biopsy specimens in 10 patients showed no evidence of a viral, infiltrative or dysplasic process in the myocardium. Baseline electrophysiological studies were performed in all patients and showed normal sinus node, atrioventricular node and His-Purkinje function in all. Inducible sustained ventricular arrhythmias occurred in 7 patients (39%). From these, 4 patients had sustained VF and 3 patients had sustained inducible monomorphic VT or ventricular flutter (cycle length 230 ± 20 ms). Two extra-stimuli were required in 5 patients and 3 extra-stimuli in 2 patients. Non-sustained VT-VF was induced in 3 patients. No arrhythmia could be induced in 8 patients.

**Table 1:** Clinical characteristics and outcome of 18 patients with idiopathic VF

**Baseline Characteristics**					**ICD Therapy**						
**Patient**	**Age (years) sex**	**CE**	**Drug test to exclude Brugada**	**PES**	**AS**	**NSVT**	**IS**	**Ind S**	**Time to 1^st ^AS (months)**	**Follow-up (months)**	**AAD during follow-up**

1	47,M	Sy-VF	F	SMVT	6	7	-	-	4	17	sotalol
2	51,F	CA	F	NI	7	21	-	-	1	19	sotalol
3	66,M	CA	AJ	VF	-	-	-	-	-	5	-
4	70,F	CA	P	NI	-	-	6(af)	-	-	19	amiodarone
5	46,M	CA	F	NI	-	1	1(af)	-	-	23	sotalol
6	20,M	Sy-VF	A	VF	-	-	3(st-lp)	-	-	92	b-blocker
7	46,F	CA	P	VF	-	120	-	-	-	27	sotalol
8	40,M	CA	A	NSVT	1	-	-	-	13	75	-
9	33,M	Sy-VF	F	NSVT	-	1	-	-	-	19	-
10	26,M	CA	F	NI	1	-	3(st-lp)	-	7	9	sotalol
11	33,F	CA	F	NI	-	-	-	-	-	18	-
12	50,F	CA	F	SMVT	6	-	1(st)	-	13	86	flecainide
13	25,M	CA	AJ + P	NSVT	-	1	-	-	-	62	-
14	36,M	CA	AJ	NI	2	-	2(af)	4	27	55	sotalol
15	58,M	Sy-VF	AJ	NI	-	-	3(af)	2	-	57	sotalol
16	44,M	CA	F + P	NI	-	-	-	-	-	48	-
17	28,M	Sy-VF	F	VF	4	-	2(st)	-	18	55	sotalol
18	35,M	Sy-VF	A	SMVT	-	-	-	-	-	59	-

CE: clinical events; CA: cardiac arrest; Sy-VF: syncope with documented self-terminating polymorphic VT or VF

PES: programmed electrical stimulation; M: male; F: female

SM: sustained monomorphic; VT: ventricular tachycardia; VF: ventricular fibrillation

NS: non sustained; NI: non inducibility; af: atrial fibrillation; st: sinus tachycardia; lp: lead problem

AS: appropriate shock; IS: inappropriate shock; Ind S: indeterminate cause for shocks;

AAD: antiarrhythmic drug; F: flécaïnide; AJ: ajmaline; P: procainamide

### Long-term outcome

After a mean follow-up of 41 ± 27 months (ranging from 5 to 92 months), 7 of the 18 patients (39%) with idiopathic VF had VF or sustained polymorphic VT recurrence and received appropriate shocks (Table 1). These patients experienced a total of 27 shocks; 5 of them had more than 1 episode (2 to 7). Mean time to first appropriate shock was 12 ± 9 months (ranging from 0.4 to 27 months). No arrhythmic storm was observed at follow-up (defined as ≥ 3 separate VT/VF episodes within 24 h). With the use of stored electrograms, 6 patients had documented non-sustained polymorphic VT or VF for a total of 149 episodes (range 1 to 120). Four of them have not received any appropriate shock. These episodes were detected by the ICD but shock delivery was appropriately aborted by the non-committed function of the device. There was no relationship between the initial clinical and arrhythmic presentation and subsequent arrhythmic events recorded from the ICD at follow-up (Table 1). From the intracardiac electrograms, the arrhythmia initiation was always associated with PVCs with a mean coupling interval of 300 ± 35 ms. A long-short initiating sequence of VF was never observed. In 5 patients, multiple VF episodes were recorded by the ICD, all of which for a single patient were associated with the same PVC coupling interval. Since the stored electrograms in the present study were obtained from endocardial sites and were single-channel recordings, we could not assess the origin of the PVCs. The mean QTc at the time of VF recurrence was normal in all patients (mean 418 ± 22 msec). The ICD effectively recognized and promptly treated all the polymorphic VT or VF recurrences and prevented the possible occurrence of sudden cardiac death. No death was reported during follow-up. Non-invasive follow-up examinations failed to detect any new structural heart disease or primary electrical disease such as long QT or Brugada syndrome.

### Value of electrophysiological Testing

Programmed electrical stimulation failed to predict subsequent cardiac events. The sensitivity and specificity were 43 and 64%, respectively. The positive and negative predictive values were also 43 and 64%, respectively, that is not considered clinically useful.

### Causes and incidence of inappropriate shocks

In this population, we observed a high incidence of inappropriate shocks. Eight of the 18 patients (44%) received an inappropriate shock for a total of 21 discharges. Atrial fibrillation was responsible for 57% of them (12 episodes in 4 patients). These were older patients with a mean age of 53 ± 14 years. Six inappropriate shocks (4 patients) were triggered by sinus tachycardia. These patients were younger with a mean age of 31 ± 13 years. Lead malfunction caused 3 inappropriate shocks in 2 patients. Six shocks in 2 patients were classified as of unknown cause, even after careful examination of the intracardiac electrograms. These shocks were probably inappropriate since no clinical symptoms occurred during these episodes and these 2 patients already experienced inappropriate shocks for atrial fibrillation.

### Device follow-up

During the follow-up period, first-time generator replacement was performed in 9 patients after a mean of 43 ± 11 months after initial implantation (range 21 to 58). End of life battery was the indication for replacement in 8 patients, and 1 had an ICD component failure. A second replacement was done in 2 patients. Lead replacement was also indicated in 2 patients for lead insulation fracture. Adjunctive anti-arrhythmic drugs were required in 11 patients after ICD implantation in order to control for the occurrence of appropriate or inappropriate shocks (Table 1).

## Discussion

To our knowledge, this report is the first study to describe the clinical outcome of ICD patients with a diagnosis of idiopathic VF in whom the Brugada syndrome, characterized by a high recurrence rate, was systematically excluded. Since there is a wide variability in the ECG expression in individual patients with the Brugada syndrome, we performed *iv *administration of sodium channel blockers to unmask the ECG features of the syndrome in all our idiopathic VF patients. Two studies reported the long-term outcome of patients with idiopathic VF without the Brugada syndrome [5,6]. Viskin et al. [5] performed *iv *administration of a sodium channel blocker to unmask a concealed form of Brugada syndrome in only 15 % of their idiopathic VF population, whereas Remme et al. [6] tested the effect of an *iv *sodium channel blocker on ECG morphology in only 30% of the patients. The unique finding of the present report is the high recurrence rate of sustained ventricular arrhythmias in this well-characterized idiopathic VF group, even after a careful systematic evaluation to exclude the presence of Brugada syndrome. After a mean follow-up of 41 ± 27 months, 39% of the patients received an appropriate ICD discharge for VF or polymorphic VT. In accordance with references [8,9], ICD therapy offered good protection against fatal outcome due to recurrent ventricular arrhythmias, with no mortality during the follow-up. The high recurrence rate is consistent with the relatively high recurrence rate of arrhythmic events or sudden death from the UCARE registry [10], as well as the high frequency of electrical discharges from ICDs reported in a large series of patients with idiopathic VF [6,8,9]. In comparison, appropriate ICD discharges have been reported in 48% of arrhythmogenic right ventricular dysplasia population [11], 40 to 56% of inducible population receiving an ICD [12,13] and 23% (7%/year) in the hypertrophic cardiomyopathy population [14]. However, some authors have reported a more benign clinical course of their idiopathic VF population with a lower recurrence rate [15-17]. Our high ICD discharge rate might also be explained by the rapid ICD intervention for fast ventricular arrhythmia that could have been self-terminated. The definition of idiopathic VF remains problematic, since numerous studies including idiopathic VF population are heterogeneous. Indeed, 61% (11/18 patients) of our cohort remained free of sustained arrhythmia recurrence during follow-up despite the same initial VF or syncopal presentation. Of note, idiopathic VF is always a diagnosis of exclusion. The patients in our cohort, as well as in other reported series, had normal hearts, as defined by clinical, non-invasive and invasive testing. Even after a careful investigation, a transient phenomenon such as a reversible localized myocardial disease, silent myocardial ischemia due to coronary artery spasm or sudden manifestation of an unknown primary electrophysiological disease could easily be missed by the clinician, and be responsible for the VF episode. Idiopathic VF is in fact an amalgam of different diseases in which the first clinical manifestation is VF. The ultimate answer probably lies in a more comprehensive approach and a more precise understanding of the molecular genetics and associated electrophysiological abnormalities in finding a specific treatment avoiding ICD implantation with its associated complications.

Anti-arrhythmic drugs have also been used in this clinical condition. Belhassen et al. [18] reported excellent results in their idiopathic VF population with EP-guided therapy using Class 1A drugs, primarily quinidine; no death occurred during a mean follow-up of >9 years. Recent evidence also suggests that frequent premature ventricular contractions arising from the Purkinje system are responsible for initiation of ventricular fibrillation, and can be mapped and ablated in selected patients [19]. Until another proven therapy has been assessed prospectively, the possibility of VF recurrence mandates ICD implantation as currently proposed in ICD guidelines [20].

### Electrophysiological findings

The inducibility rate (39%) of sustained VT/VF observed in our study is also in accordance with the results of the UCARE registry [10]. Of importance, programmed electrical stimulation was of limited value with a poor sensitivity and specificity. Moreover, inducibility failed to predict subsequent arrhythmic events. Our findings differ from the observed 79% average inducibility rate reported by Belhassen et al. [17,18]. This may be due to different patient characteristics and stimulation protocol. Our study excluded patients with Brugada syndrome known to have a high inducibility rate [21]. Compared to our stimulation protocol, Belhassen et al. [22] used up to 3 extra-stimuli, 2 basic cycle lengths, 2 RV sites (first RVA, then RVOT), and repetition of extra-stimulation (*n *= 10 for double, and *n *= 5 for triple) at the shortest coupling intervals that resulted in ventricular capture. Three patients had a fast monomorphic VT inducible even if the clinical presentation was aborted sudden cardiac death. This may suggest an underlying structural heart disease undetected by the current investigation. Although general cardiac function can be normal, patients might have discrete abnormalities, which are currently unidentifiable. Better risk stratification is required to identify patients who will experience recurrent VF over time. Therefore, defibrillator implantation could be seen as the primary therapy in patients with idiopathic VF, since no stratification has yet identified patients at risk of arrhythmia recurrence.

### ICD limitations

The impact of multiple battery replacements over time in this young population with idiopathic VF needs to be emphasized. It is noteworthy that half the population (9/18 patients) had an ICD replacement and 2 other patients had their transvenous lead replaced during follow-up. We also observed a high incidence of inappropriate shocks (44%), mainly caused by atrial fibrillation and sinus tachycardia. This is well in accordance with other studies where up to 40% inappropriate discharge rates were observed even with fourth generation ICDs [23]. The addition of an anti-arrhythmic drug to decrease the incidence of inappropriate shocks was required in this group. Four patients experienced atrial fibrillation, which might indicate the presence of an associated primary electrophysiological disease also affecting the atrium. In patients with idiopathic VF, the characteristics, is the recurrence of VF and not monomorphic VT compared to other ventricular arrhythmias. To decrease the number of inappropriate shocks, one should program a higher VF zone around 200–220 bpm and a lower monitoring zone to detect atrial arrhythmia. Third-generation ICD still have limitations and complications over time, with a significant proportion of patients having hardware-related complications or inappropriate shocks [24]. Future developments in ICD technology is needed, which will hopefully address these issues.

## Conclusions

Idiopathic VF patients have a high recurrence rate of ventricular arrhythmias, even after the systematic exclusion of patients with the Brugada syndrome or other known electrical diseases from the analysis. ICD prevents sudden cardiac death in this population, but treat only the final manifestation of an unknown disease. Inappropriate shocks remain a major concern in this young population.

## Competing interests

The author(s) declare that they have no competing interests.

## Author's contributions

JC participated in the hypothesis generation and drafted the manuscript. PG and PB were project managers. FP participated in the final writing of the manuscript. All authors have read and approved the final version of the manuscript.

## Pre-publication history

The pre-publication history for this paper can be accessed here:


